# Impact of a primary care pharmacist consultations on pregnant women’s medication use: the SafeStart intervention study linked to a national prescription database

**DOI:** 10.1007/s11096-023-01577-x

**Published:** 2023-05-08

**Authors:** Elin Ngo, Maria Bich-Thuy Truong, Hedvig Nordeng

**Affiliations:** 1 PharmacoEpidemiology and Drug Safety, Department of Pharmacy, University of Oslo, Blindern, Postbox 1068, 0316 Oslo, Norway; 2 Department of Child Health and Development, National Institute of Public Health, Oslo, Norway

**Keywords:** Community pharmacy, Intervention, Medication, NorPD, NVP, Pharmaceutical care, Pharmacist counseling, Pregnancy, Prenatal care, Prescription database, SafeStart

## Abstract

**Background:**

Prior studies show that pharmacist consultations are highly appreciated by pregnant women and feasible in community pharmacies. However, it is unknown whether such counseling has an impact on medication use during pregnancy.

**Aim:**

This study aimed to assess whether a pharmacist consultation in early pregnancy was associated with pregnant women’s medication use, with a focus on antiemetic medications.

**Method:**

The SafeStart study recruited Norwegian pregnant women in the first trimester between February 2018 and February 2019. Women in the intervention group received a pharmacist consultation in a community pharmacy or by phone. A follow-up questionnaire was completed 13 weeks after enrollment. Data from the SafeStart study were linked to the Norwegian Prescription Database. Logistic regression was used to assess the association between the pharmacist intervention and medication use in the second trimester.

**Results:**

The study included 103 women in the intervention group and 126 in the control group. Overall prescription fills in the first and second trimesters were 55% and 45% (intervention group) and 49% and 52% (control group), respectively. In total, 16–20% of women in the first trimester and 21–27% of women in the second trimester had a prescription for antiemetics. The pharmacist intervention was not associated with women’s medication use in the second trimester.

**Conclusion:**

This study did not detect an impact of a pharmacist consultation on pregnant women’s use of medications. In the future, pharmacist consultations should focus on other outcome factors, such as risk perception, knowledge level, and the use of other health care services.

**Trial registration** The SafeStart study is registered with ClinicalTrials.gov (identifier: NCT04182750, registration date: December 2, 2019).

**Supplementary Information:**

The online version contains supplementary material available at 10.1007/s11096-023-01577-x.

## Impact statements

- Information about advice for, and treatment of, pregnancy-related conditions is highly requested. Available information should be easily accessed by pregnant women.
- Although a pharmacist consultation did not impact medication use during pregnancy, it is still unknown whether the role of pharmacists in maternity care may benefit pregnant women’s medication use.
- Intervention studies among pregnant women need to take into account women of high socioeconomic status when estimating the effect of an intervention.

## Introduction

Up to 90% of pregnant women use medications during pregnancy [1, 2]. The use of prescribed and over-the-counter medications in the first trimester has increased by more than 60% in the last three decades [3]. Despite widespread use, pregnant women still report a lack of information from their health care providers regarding safe medication use during pregnancy [4], including for the treatment of nausea and vomiting in pregnancy (NVP) [5].

NVP affects up to 80% of pregnant women and often starts around gestational weeks 4–9 [6–8]. Although safe pharmacological treatments for NVP are available [9–12], the combination of trivializing NVP, lack of knowledge about the use of antiemetic medications during pregnancy, and fears of fetal harm often lead to late recognition and undertreatment of NVP [13, 14].

Moreover, up to 77% of pregnant and postpartum women report the need for information regarding the use of medications during pregnancy [15]. Even though pregnant women frequently use the internet to search for information about medication use [16, 17], they prefer to receive this information from their health care providers, such as GPs, midwives, and pharmacists [4].

Pharmacists are an important information source for pregnant women with respect to OTC medications and the management of minor ailments during pregnancy [18]. Patient-centered consultations have led to increased knowledge, compliance and enhanced health outcomes among pregnant women [19]. We previously found that a pharmacist consultation for pregnant women in the first trimester was feasible and highly appreciated by the women [20]. Women found it most useful when the information they received was tailored to their needs and when the consultations could be performed over the phone [20, 21]. However, these studies did not explore the impact of pharmacist consultations on medication use during pregnancy.

### Aim

We hypothesized that a pharmacist consultation in the first trimester of pregnancy could impact the extent and type of medications used in the second trimester. Therefore, the aim of this study was to assess whether a community pharmacist consultation in early pregnancy is associated with women’s medication use in the second trimester, with a particular focus on antiemetic medications.

### Ethics approval

The SafeStart project was approved by the Regional Committees for Medical and Health Research Ethics in Norway on November 23, 2016 (Reference: 2016/1686).

## Method

### The SafeStart study

This study was a part of the SafeStart interventional trial [20, 21]. Norwegian-speaking pregnant women in their first trimester were eligible for participation. The SafeStart interventional trial included a total of 229 women who responded to the baseline questionnaire (Q1) and follow-up questionnaire (Q2). These women were included in the analyzes of this study. Of the 229 women, 103 were allocated to the intervention group and 126 were allocated to the control group. The SafeStart study was conducted according to the CONSORT guidelines [22].

#### Recruitment

For the SafeStart interventional trial, pregnant women were recruited between February 2018 and February 2019 through Facebook (i.e., our own Facebook page for the study), pregnancy-related webpages/forums (e.g., “*altformamma.no*”, and “*tryggmammamedisin.no*), and flyers in pharmacies throughout Norway.

#### Sample size

Power analysis performed in the SafeStart interventional trial estimated that a sample size of 385 pregnant women would be needed to detect a medium effect size (Cohen’s d = 0.5) based on a two-sided α of 0.05, a power of 80%, and a dropout rate of 30%. We then performed a post hoc power analysis to determine our actual study power. Post hoc power analysis showed that a sample size of 229 (complete cases) from the SafeStart interventional trial was sufficient to detect a 19% difference in medication use with 80% power.

#### Allocation

All women who consented to participate were assigned (1:1) to either the intervention group or the control group by software developed specifically for this project. The software automatically handled the women’s enrollment, group allocation, and distribution of informational emails and online questionnaires.

#### The intervention group

The women in the intervention group received a tailored pharmacist consultation at one of the 14 pharmacies that voluntarily participated in the study or over the phone. The consultation lasted up to 15 min. The pharmacist conducting the consultation had access to the women’s answers to Q1 in advance. This information was used to prepare a structured, individualized consultation that addressed each woman’s concerns and needs.

#### The control group

Women assigned to the control group received only standard Norwegian prenatal care. Prenatal care in Norway is offered to all Norwegian pregnant women, and the basic program consists of nine consultations in total, with the first consultation recommended in gestational weeks 6–12. Prenatal care is free of charge [23].

### Data collection

#### SafeStart survey data

**Fig. 1:**
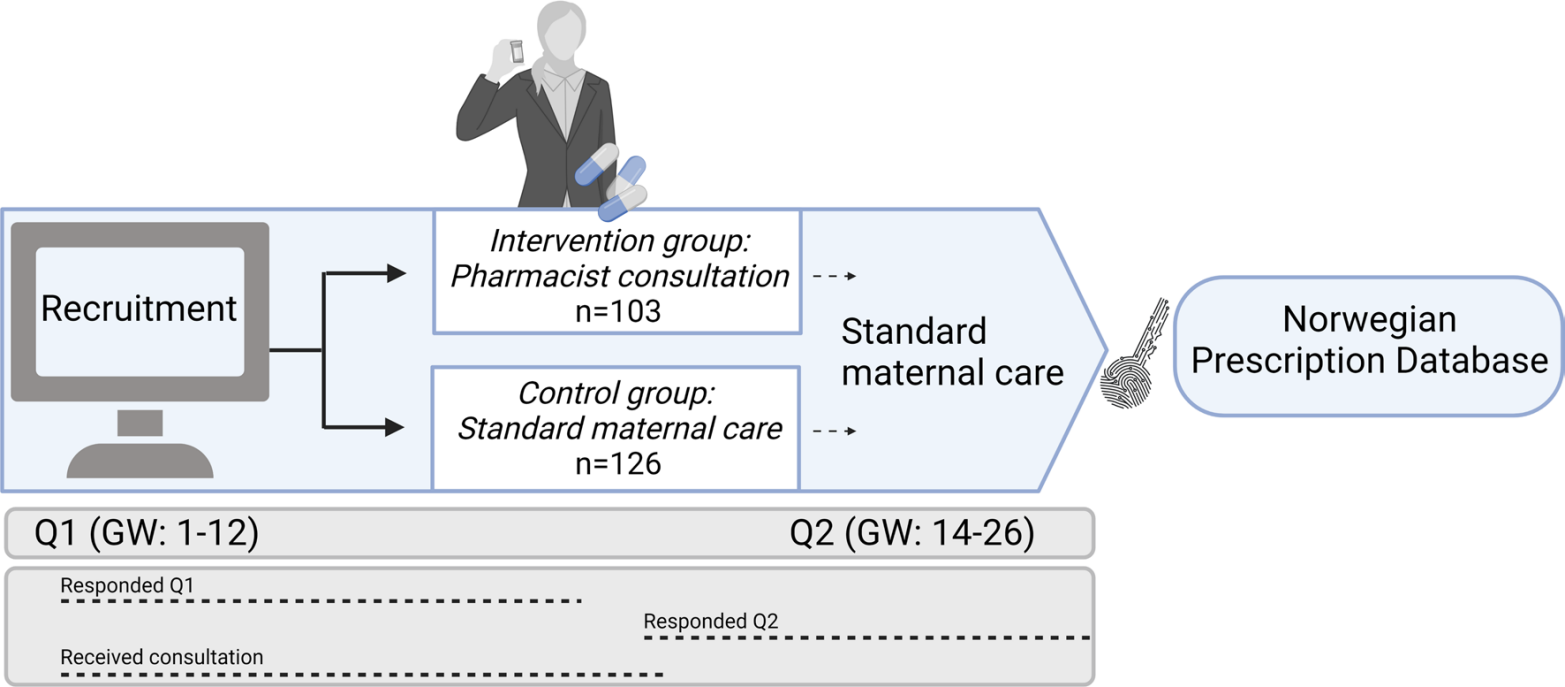
Overview of the SafeStart study design. Pregnant women were mainly recruited through social media and allocated to either the intervention or control groups. Women in the intervention group were offered a tailored pharmacist consultation. All women received standard maternal care. The women responded to Q1 and Q2 between GW 3–13 and GW 14–26, respectively. The pharmacist consultations were performed from GW 4–14 for women in the intervention group. One woman received the intervention in GW 17. Self-reported data from the SafeStart questionnaires (Q1 and Q2) were linked to data from the Norwegian Prescription Database (NorPD) by using the women’s unique social security numbers. *GW* gestational week, *Q1* baseline questionnaire, *Q2* follow-up questionnaire. (*Created with BioRender.com*)

The SafeStart interventional trial included four sets of questionnaires (Q1-Q4). In this study, we analyzed data from Q1, Q2, and the study pharmacists’ notes from the consultation. Q1 was completed at enrollment in the first trimester, and Q2 was distributed 13 weeks after enrollment and completed in the second trimester (Fig. 1).

#### SafeStart survey data: Q1

Q1 included questions about the women’s sociodemographic and lifestyle characteristics, chronic conditions and NVP severity. Q1 also included a list of health conditions (e.g., allergies, general pain, heartburn, NVP, constipation) and related medication use.

#### SafeStart survey data: Q2

The follow-up questionnaire, Q2, was distributed 13 weeks after enrollment and aimed to identify medication use in the second trimester, defined as gestational weeks 14–26. Q2 included the repeated list of medical conditions and related medication use from Q1. The English versions of Q1 and Q2 are provided in Supplementary file 1.

#### SafeStart survey data: pharmacist notes

Pharmacist notes provided information about the consultation, such as the setting and duration, in addition to the topics discussed and pregnancy-related conditions addressed during the consultation.

#### Prescription registry data

The SafeStart survey data were linked to the NorPD data by the women’s unique social security numbers. NorPD is a national registry covering all prescribed medications dispensed at pharmacies to individual patients in Norway. NorPD data include the medication name, ATC code, defined daily dose, package size, and dispense date for the participant. The completion date of Q1 and the reported gestational week reported in Q1 were used to calculate the pregnancy start date. The three months before the start of pregnancy was defined by the pregnancy start date minus 90 days. The trimesters were defined as follows: first trimester: 1–90 days after the pregnancy start date; second trimester: 91–180 days after the pregnancy start date; and third trimester: 180 days after the pregnancy start date and until delivery. Three months postpartum was defined as the estimated date of delivery plus 90 days. The time point of medication exposure during the pregnancy period, which included the three months before the pregnancy start date and three months postpartum, was identified by utilizing the dispensing date as registered in the NorPD.

#### Data storage

All collected data were stored and analyzed at the Service for Sensitive Data at the University of Oslo (TSD) [24]. The TSD is protected by two-factor authentication and designed for storing and postprocessing sensitive data in compliance with the Norwegian *“Personal Data Act”*, “Health Research Act”, and regulations regarding an individual’s privacy.

The datasets used in this study are from a third party and are not publicly available due to ethical and legal restrictions. Please contact the corresponding author for further information regarding the questionnaires and the data.

### Outcome measures: medication use

The outcome measure was medication use in the second trimester. The outcome was assessed by evaluating the differences in medication use in the second trimester among women in the intervention and control groups.

All medications were classified in the anatomical/pharmacological group by the Anatomical Therapeutic Chemical (ATC) classification system (ATC 1st level) [25]. Antiemetics were classified at the substance level (ATC 5th level).

### Statistical methods

#### Descriptive analyzes

All analyzes were performed as complete case analyzes. Complete case analyzes was chosen because it reflects the ideal situation of the impact of a pharmacist consultation on medication use during pregnancy.

We compared the baseline characteristics of the intervention and control groups to evaluate whether the allocation process produced balanced groups. This was also done to evaluate covariate balance, as complete case analyzes put the randomization process at risk. The chi-squared test was used to compare categorical variables, i.e., relationship status, education level, work situation, folic acid supplementation, parity, pregnancy-related conditions, and chronic conditions, which are presented as medians and ranges. Student’s test was used to compare continuous variables, i.e., gestational week, maternal age, and Pregnancy Unique Quantification of Emesis (PUQE) score, which are presented as counts and percentages. Proportions of filled prescriptions of medications and their ATC codes with at least 20 women in the defined time periods as registered in the NorPD were calculated for the five pregnancy periods: three months before pregnancy, the first, second, and third trimesters, and three months postpartum. Filled prescriptions for antiemetic medications were considered for the first and second trimesters only.

#### Association analyzes

Logistic regression was performed to estimate the association between pharmacist consultations (intervention vs. control groups) and second trimester medication use. Separate models were computed for self-reported medication use and filled prescriptions, including medications in general and antiemetic medications specifically. The results are presented as crude and adjusted odds ratios (ORs) with 95% confidence intervals (CIs). The adjusted ORs were adjusted for medication use in the first trimester and employment status at baseline, as these variables were unbalanced between the intervention and control groups at baseline.

#### Sensitivity analysis

We performed a predefined stratified analysis according to employment status to assess effect modification by being employed as a health care worker. We hypothesized that the intervention would have a different impact on medication use among pregnant women working as health care workers compared to those working elsewhere, as we assumed that health care workers would have a higher knowledge level regarding health care and medication use.

## Results

### Study population

**Fig. 2:**
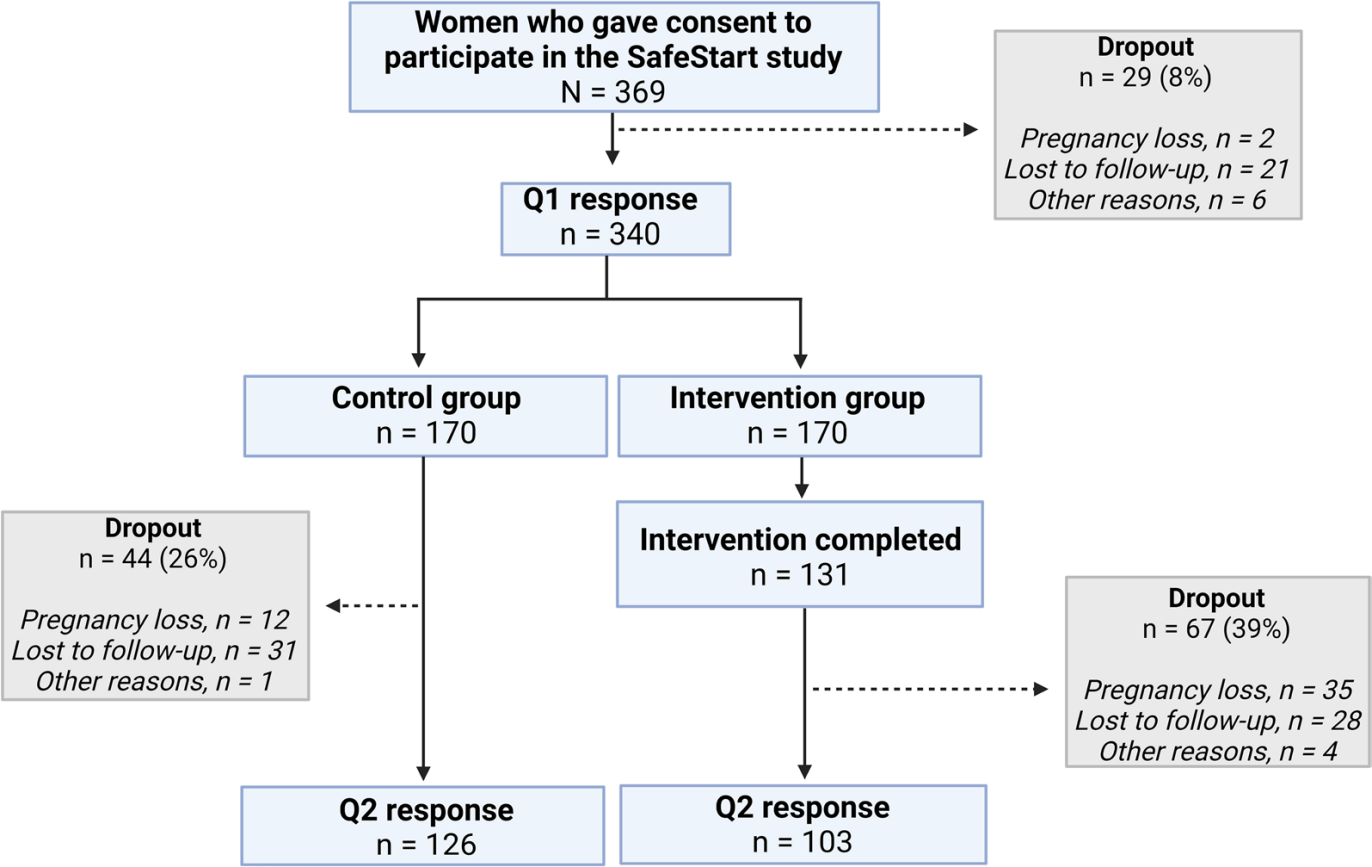
Flowchart of the SafeStart inclusion and exclusion criteria to determine the final study population. A total of 369 women gave consent to participate in the study, which resulted in 103 women in the intervention group and 126 women in the control group. All analyzes were performed as complete case analyzes (N = 229). *Q1* Baseline questionnaire. *Q2* Follow-up questionnaire. (*Created with BioRender.com*)

In total, 103 pregnant women were allocated to the intervention group and 126 to the control group (Fig. 2). The median gestational week at enrollment was week 7 (intervention group range: 3–12 weeks; control group range: 3–13 weeks). The mean PUQE score for both groups was 6 points (range: 3–14 and 3–15) at baseline, and half of the pregnant women scored > 6 points. There was a significant difference in employment status between the two study groups (chi-square test, *p* = *0.03)*. The study population baseline characteristics are presented in Table 1.

**Table 1:** Baseline characteristics of the study population according to the study groups (intervention group, n = 103 and control group, n = 126)

Characteristics	n	Intervention group (n = 103)	n	Control group (n = 126)	Balance of covariates (p value)*
		Value (median, range or %)		Value (median, range or %)	
Gestational week at enrollment		7 (3–12)		7 (3–13)	0.65
Maternal age (years)		31 (21–40)		31 (21–41)	0.65
Relationship status					0.48
Married/cohabitating	100	97.1	121	96.1	
Single	3	2.9	5	3.9	
Higher education					0.42
Yes	89	86.4	105	83.3	
No	14	13.6	21	16.7	
Employment status					0.02
Employed	71	68.9	63	50.0	
Employed in the health sector	23	22.3	39	30.9	
Other	9	8.8	24	19.1	
Primigravida					0.22
Yes	64	62.1	61	48.4	
No	39	37.9	65	51.6	
Folic acid supplementation before/during pregnancy					0.23
Yes	102	99.1	124	98.4	
No	1	0.9	2	1.6	
PUQE score		6 (3–14)		6 (3–15)	0.40
Chronic conditions					
Asthma	9	8.7	15	11.9	0.44
Allergies	20	19.4	32	25.4	0.28
Hypothyroidism	4	3.9	6	4.8	0.75
Depression/anxiety	7	6.8	9	7.1	0.92
Other**	19	18.4	26	20.6	0.50

*n* number of women, *SD* standard deviation, *PUQE score* Pregnancy Unique Quantification of Emesis score

^*^Chi-square tests were used to compare categorical variables, and Student’s t tests were used to compare continuous variables

^**^Other chronic conditions include ADHD, cardiovascular disease, chronic fatigue syndrome, Crohn’s disease, eczema, endometrioses, epilepsy, fibromyalgia, high cholesterol, hyperthyroidism, irritable bowel syndrome, mental disorders, migraine, multiple sclerosis, polycystic ovary syndrome, psoriasis, rheumatic diseases, sarcoidosis, and ulcerative colitis

### The intervention

Of 103 pharmacist consultations, 37 (36%) were performed at the study pharmacies and 66 (64%) were performed over the phone. All consultations were performed between gestational weeks 4–14. One woman received the consultation in week 17, but still prior to the completion of Q2. The most frequent topics addressed during the consultations were *advice and treatment of pregnancy-related conditions* (61/103, 59%). NVP was the pregnancy-related condition that was most frequently addressed during pharmacist consultations (49/103, 48%) (Supplementary file 2).

### Medication use

#### Self-reported medication use (SafeStart study data)

Women in the intervention and control groups most frequently self-reported having used medications with ATC codes A *(alimentary tract and metabolism*), N (*nervous system)*, and R *(respiratory system)*. Both groups reported having used medications with ATC codes A and N more frequently in the second trimester (ATC code A: 20–25% and N: 45–47%) than in the first trimester (ATC code A: 7–8% and N: 6–8%, Table 2).

**Table 2:** Overview of the number of women with filled prescriptions as registered in the Norwegian Prescription Database (NorPD) and self-reported medication use in the baseline (Q1) and follow-up questionnaires (Q2)

	Intervention group	Control group	Intervention group	Control group	Impact of a pharmacist consultation on medication use in the 2nd trimester	
	Medication use in the 1st trimester	Medication use in the 1st trimester	Medication use in the 2nd trimester	Medication use in the 2nd trimester		
	n (%)	n (%)	n (%)	n (%)	Crude OR (95% CI)	Adjusted OR* (95% CI)
Filled prescriptions as registered in the NorPD****						
A: Alimentary tract and metabolism	29 (28.2)	37 (29.4)	24 (23.3)	34 (26.9)	0.8 (0.4, 1.5)	0.7 (0.3, 1.7)
B: Blood and blood forming organs	15 (14.6)	21 (16.7)	16 (15.5)	26 (20.6)	0.7 (0.4, 1.4)	0.6 (0.2, 1.9)
G: Genito-urinary system and sex hormones	37 (35.9)	51 (40.5)	23 (22.3)	48 (38.1)	0.5 (0.2, 0.8)	0.4 (0.2, 0.8)
H: Systemic hormonal preparations	16 (15.5)	28 (22.2)	13 (12.6)	26 (20.6)	0.6 (0.3, 1.1)	0.6 (0.2, 1.9)
J: Anti-infectives for systemic use	41 (39.8)	41 (32.5)	36 (34.9)	47 (37.3)	0.9 (0.5, 1.6)	0.7 (0.3, 1.3)
N: Nervous system	18 (17.5)	35 (27.8)	14 (13.6)	31 (24.6)	0.5 (0.2, 0.9)	0.6 (0.2, 1.6)
R: Respiratory system	36 (34.9)	39 (30.9)	32 (31.1)	39 (30.9)	1.0 (0.6, 1.8)	0.8 (0.4, 1.7)
Total***	57 (55.3)	62 (49.2)	46 (44.7)	65 (51.6)	0.8 (0.4, 1.3)	0.7 (0.4, 1.2)
Self-reported medication use**						
A: Alimentary tract and metabolism	7 (6.8)	10 (7.9)	21 (20.4)	32 (25.4)	0.8 (0.4, 1.4)	0.8 (0.4, 1.5)
N: Nervous system	6 (5.8)	10 (7.9)	46 (44.6)	59 (46.8)	0.9 (0.5, 1.5)	1.0 (0.6, 1.7)
R: Respiratory system	25 (24.3)	34 (26.9)	28 (27.2)	40 (31.7)	0.8 (0.5, 1.4)	0.8 (0.4, 1.5)
Total****	36 (34.9)	45 (35.7)	59 (57.3)	83 (65.9)	0.7 (0.4, 1.2)	0.7 (0.4, 1.2)

The impact of a pharmacist consultation on medication use in the second trimester in the intervention (N = 103) and control groups (N = 126) are presented as crude ORs and adjusted ORs

*NorPD* Norwegian Prescription Database, *n* Number of women

^*^Adjusted for medication use and employment status at baseline

^**^ATC codes S (sensory system) and M (musculoskeletal system) are not included in this table because the number of women who reported them was below 10

^***^Total number of women who reported at least one medication/ had at least one filled prescription registered in the NorPD

^****^ATC codes C (cardiovascular system), D (dermatologicals), L (antineoplastic and immunomodulating agents), M (musculoskeletal system), P (antiparasitic products, insecticides and repellents), S (sensory system), and V (various) were not included in this table as the number of women who reported them was below 10

#### Filled prescriptions (prescription registry data)

The most commonly filled prescriptions for both groups were for medications with ATC codes A, G (*genito-urinary system and sex hormones),* J (*anti-infectives for systemic use),* and R. The rates of filled prescriptions with each ATC code were similar in all periods for both study groups (Table 2 and Supplementary file 3).

### Associations between the pharmacist intervention and medication use in the second trimester

#### Self-reported medication use (SafeStart study data)

No differences were detected in self-reported medication use in the second trimester between the intervention and control groups for ATC codes A (adjusted OR (aOR): 0.8, 95% CI: 0.4, 1.5), N (aOR: 1.0, 95% CI: 0.6, 1.7) or R (aOR: 0.8, 95% CI: 0.4, 1.5). The analyses are presented in Table 2.

#### Filled prescriptions (prescription registry data)

There was no difference between the intervention and control groups in filled prescriptions during the second trimester, except for medications with ATC code G, where women in the intervention group had lower odds of filling prescriptions after the pharmacist consultation (aOR: 0.4, 95% CI: 0.2, 0.8, Table 2).

#### Prescribed antiemetic medications (Prescription registry data)

A total of 28 women in the intervention group and 27 women in the control group had filled a prescription for an antiemetic medication in the second trimester (Table 3). However, there was a lower, but not significant, difference in the number of filled prescriptions for antiemetic medications in the second trimester between the two study groups (aOR: 0.4, 95% CI: 0.1, 1.4).

**Table 3:** Overview of filled prescriptions for antiemetic medications in the intervention (N = 103) and control groups (N = 126) as registered in the Norwegian Prescription Database (NorPD) in the 1st (T1) and 2nd trimester (T2)

Antiemetic medication	Intervention group	Control group	Intervention group	Control group	Use of antiemetic medications during the 2nd trimester	
	T1 n (%)	T1 n (%)	T2 n (%)	T2 n (%)	Crude OR (95% CI)	Adjusted OR* (95% CI)
Meclizine	9 (8.7)	6 (4.8)	5 (4.9)	4 (3.2)	–	–
Promethazine	3 (2.9)	8 (6.3)	3 (2.9)	7 (5.6)	–	–
Metoclopramide	9 (8.7)	6 (4.8)	20 (19.4)	16 (12.7)	–	–
Total*	21 (20.4)	20 (15.9)	28 (27.2)	27 (21.4)	0.6 (0.3, 1.2)	0.4 (0.1, 1.4)

The impact of an early pharmacist consultation on the use of antiemetic medications in the second trimester in the intervention and control groups is presented as crude ORs and adjusted ORs

*T1* First trimester, *T2* Second trimester, *n* number of women

^*^Adjusted for medication use and employment status at baseline

In the analysis stratified by employment status, we found lower odds of filled prescriptions for antiemetic medications among women who were employed in the health care sector than among women employed in other sectors (aOR: 0.3, 95% CI: 0.2, 0.5).

## Discussion

### Main results

To our knowledge, this study is the first to assess the impact of a pharmacist consultation in the first trimester on medication use during pregnancy. This study showed no association between pharmacist consultations and the use of medications in the second trimester of pregnancy.

In comparison to an earlier multinational study [2] and similar to a Swedish register-based study [26], in our study, pregnant women also filled medications with ATC codes A, J, N, and R as the most frequently used medications. In line with other Scandinavian studies, medications with ATC code J were the most frequently prescribed medications for pregnant women [26–28]. In the second trimester, 27% of the women in the intervention group and 21% in the control group had filled prescriptions for antiemetics. This is considerably higher than the rate in a previous Norwegian registry study (2005–2017) that showed that 8% of pregnant women filled at least one prescription for antiemetics during pregnancy [29]. Given that approximately half of the women in the SafeStart study received a score over the cutoff value for moderate NVP (≥ 6 points) and that 48% of the women in the intervention group discussed NVP during the consultation, the higher number of prescribed antiemetic medications is therefore reasonable. The Norwegian registry study reported meclizine, promethazine, and metoclopramide as the most commonly prescribed antiemetic medications [29], which aligns well with our study.

The lack of association between pharmacist consultations and medication use during pregnancy may be due to several reasons. Our study population was a more resourceful group of women with higher education than the general birthing population in Norway (Supplementary file 4). Over half of the women in the study were primiparous and were more likely to actively seek medical information online [16, 17, 30, 31]. It is possible that well-informed groups of women benefit less from pharmacist consultations than less resourceful groups of women. Other studies have shown that pregnant women trust pharmacists to provide them with information about medications [15, 16, 32]. We cannot exclude the possibility that women in the control group became aware of the type of information available and contacted other pharmacies outside of their study participation.

### Strengths and limitations

The main strength of this study was that we were able to recruit women from all parts of Norway, consequently increasing the generalizability of our results beyond one study site. Another strength of this study was the linking of self-reported data regarding medication use to filled prescriptions as recorded in the NorPD.

Limitations to take into consideration are selection bias and recall bias. Our study included a resourceful group of women with higher educational status. As in other studies based on the recruitment of women, there is always an inherent risk of selection bias toward more interested and motivated individuals. Moreover, the data on medication use collected in Q1 and Q2 were self-reported, which may introduce recall bias; for example, women in the intervention group may have provided more accurate reports than those in the control group. This bias, however, would not be present in the analyses based on data from the prescription registry, as registry data were recorded independent of the intervention. Another limitation to consider is that Q1 and Q2 did not include identical lists of medical conditions and related medications. Therefore, self-reported medication use may not be directly comparable to illnesses but only to medication use in general.

### Future research

Future work should focus on the role of pharmacies within maternity care. The most frequent pregnancy-related condition addressed during the consultations was NVP. This indicates that the role of pharmacists may be beneficial for women with pregnancy-related symptoms that occur in early pregnancy, often prior to their first prenatal care visit [8, 33]. Moreover, future studies should investigate the impact of a pharmacist consultation on other outcomes that are equally important for women’s daily lives, such as knowledge about medication use, risk perception, and the utilization of health care services.

Moreover, digitalization and m- and eHealth have all been shown to be beneficial as a part of patient care. In particular, mobile applications, websites, and other digital programs for improving health and medication use in pregnant women have been shown to be beneficial [34–37]. There has been a call for digital technologies to promote self-care and improve communication between pregnant women and health care providers [38, 39]. Future studies should therefore explore how pharmacists use digital tools as a part of pharmaceutical care.

## Conclusion

In this study, we did not detect an impact of an early pharmacist consultation on medication use in general or the use of antiemetic medications during pregnancy. The results may have been affected by the study population, which included a large proportion of women with high socioeconomic status. Future studies should focus on the impact of pharmacist consultations on other outcome factors and the role of pharmacists in maternity care.

## Supplementary Information

Below is the link to the electronic supplementary material.

Supplementary file1 (PDF 533 KB)

Supplementary file2 (PDF 186 KB)

Supplementary file3 (PDF 195 KB)

Supplementary file4 (PDF 284 KB)
