# Supplementary material for: Impact of a primary care pharmacist consultations on pregnant women’s medication use: the SafeStart intervention study linked to a national prescription database
Source: Int J Clin Pharm. 2023 May 8;45(4):893–902. doi: 10.1007/s11096-023-01577-x (PMC10366231; doi:10.1007/s11096-023-01577-x)
Supplement: Supplementary file 1 — Supplementary file1 (PDF 533 KB) [file 11096_2023_1577_MOESM1_ESM.pdf]

SafeStart Q1: Gestational weeks 1-12

INFORMATION ABOUT YOURSELF

1. **What is your social security number (11 digits)?**
2. **How many children do you already have?**
    a. None
    b. One
    c. Two
    d. More than two
3. **What is your relationship status?**
    a. Married
    b. Cohabitating
    c. Single
    d. Divorced/separated
    e. Other
4. **What is your highest educational degree?**
    a. Secondary school
    b. High school
    c. College/University
    d. Other
5. **What is your work situation?**
    a. Student
    b. Stay at home
    c. Employed in the health sector
    d. Employed
    e. Job seeker
    f. None of the above

INFORMATION ABOUT YOUR PREGNANCY

6. **What is your current gestational week?** (Specify)
7. **Have you been pregnant before?**
    a. Yes
    b. No
8. **Are you pregnant with more than one child?**
    a. Yes
    b. No
    c. I don't know
9. **On average in a day, for how long do you feel nauseated or sick to your stomach?**
    a. More than 6 hours
    b. 4-6 hours
    c. 2-3 hours
    d. 1 hour or less
    e. Not at all
10. **On average in a day, how many times do you vomit or throw up?**
    a. More than 7 times
    b. 5-6 times
    c. 3-4 times
    d. 1-2 times
    e. Not at all
11. **On average in a day, how many times have you had retching or dry heaves without bringing anything up?**
    a. More than 7 times

b. 5-6 times
c. 3-4 times
d. 1-2 times
e. Not at all

12. Have you taken folic acid/folate during this pregnancy?
a. Yes, before the pregnancy
b. Yes, before and during the pregnancy
c. Yes, during the pregnancy only
d. No
e. I don't recall

PREGNANCY-RELATED AILMENTS AND MEDICATION USE

13. Have you experienced any pregnancy-related ailments during this pregnancy?
a. Yes
b. No

Question 14 is only available if the woman ticks off having any pregnancy-related ailments.

14. Which of the ailments below have you experienced during this pregnancy?
a. Nausea and vomiting
b. Heartburn
c. Constipation
d. Common cold and/or stuffy nose
e. Urinary tract infection
f. Other infections
g. Pain in the back, neck and/or pelvis
h. Headache
i. Sleep problems
j. Other (Specify)

Questions 15-21 are only available if the woman ticks off any of the related ailments listed above.

15. Please select which medicine(s) you have used for nausea and/or vomiting during this pregnancy.
a. Meclizine
b. Promethazine
c. Metoclopramide
d. Ondansetron
e. Prochlorperazine
f. Other
g. I have not used medicines for nausea and/or vomiting during this pregnancy

16. Please select which medicine(s) you have used for heartburn during this pregnancy.
a. Alginic acid
b. Magnesium hydroxide
c. Ranitidine
d. Omeprazole
e. Other
f. I have not used medicines for heartburn during this pregnancy

17. Please select which medicine(s) you have used for constipation during this pregnancy.
a. Lactulose
b. Ispaghula
c. Bisacodyl
d. Sennoside a+b
e. Sodium picosulfate
f. Klyx or Microlax
g. Macrogol
h. Other

i. I have not used medicines for constipation during this pregnancy

18. Please select which medicine(s) you have used for the common cold and/or stuffy nose during this pregnancy.

a. Xylometazoline
b. Xylometazoline and ipratropium bromide
c. Noscapine
d. Ethylmorphine
e. Codeine
f. Bromhexine
g. Guaifenesin
h. Other
i. I have not used medicines for the common cold and/or stuffy nose during this pregnancy

19. Please select which medicine(s) you have used for urinary tract infections during this pregnancy.

a. Pivmecillinam
b. Nitrofurantoin
c. Trimethoprim (+Sulfamethoxazole)
d. Amoxicillin
e. Other
f. I have not used medicines for urinary tract infections during this pregnancy

20. Please select which medicine(s) you have used for pain in the back, neck and/or pelvis during this pregnancy.

a. Paracetamol
b. Ibuprofen
c. Naproxen
d. Diclofenac
e. Codeine and paracetamol
f. Morphine
g. Other
h. I have not used medicines for pain in the back, neck and/or pelvis during this pregnancy

21. Please select which medicine(s) you have used for sleep problems during this pregnancy.

a. Zopiclone
b. Promethazine
c. Alimemazine
d. Zolpidem
e. Other
f. I have not used medicines for sleep problems during this pregnancy

CHRONIC DISEASES AND MEDICATION USE

22. Do you have any chronic diseases?

a. Yes
b. No

Question 23 is only available if the woman ticks off having any chronic diseases.

23. Please tick off whether you have any of the following chronic diseases listed below.

a. Asthma
b. Allergies
c. Hypothyroidism
d. Rheumatic diseases (including rheumatoid arthritis and psoriatic arthritis)
e. Type 1 or 2 diabetes
f. Epilepsy
g. Depression and/or anxiety
h. Cardiovascular diseases
i. Other (please specify which other disease)

Questions 24-31 are only available if the woman ticks off any of the related chronic diseases listed above.

24. If you use medication for asthma, please select the medications you use.

a. Salbutamol
b. Terbutaline
c. Salmeterol
d. Fluticasone
e. Budesonide
f. Ciclesonide
g. Ipratropium bromide
h. Other
i. I have not used medicines for asthma during this pregnancy

25. If you use medication for allergies, please select the medications you use.

a. Desloratadine
b. Cetirizine
c. Ebastine
d. Loratadine
e. Fexofenadine
f. Nasal spray and/or eye drops only
g. Other
h. I have not used medicines for allergies during this pregnancy

26. If you use medication for hypothyroidism, please select the medications you use.

a. Levothyroxine
b. Liothyronine
c. Combination of Levothyroxine and Liothyronine
d. Other
e. I have not used medicines for hypothyroidism during this pregnancy

27. If you use medication for rheumatic diseases, please select the medications you use.

a. Ibuprofen
b. Paracetamol
c. Phenazone + codeine
d. Tramadol
e. Prednisolone
f. Methotrexate
g. biological medications (given as injections)
h. Other
i. I have not used medicines for rheumatic diseases during this pregnancy

28. If you use medication for type 1 or 2 diabetes, please select the medications you use.

a. Insulin
b. Metformin
c. Glimepiride
d. Sitagliptin
e. Other
f. I have not used medicines for type 1 or 2 diabetes during this pregnancy

29. If you use medication for epilepsy, please select the medications you use.

a. Carbamazepine
b. Oxcarbazepine
c. Valproic acid
d. Lamotrigine
e. Topiramate
f. Levetiracetam
g. Other
h. I have not used medicines for epilepsy during this pregnancy

30. If you use medication for depression and/or anxiety, please select the medications you use.

a. Escitalopram
b. Citalopram
c. Sertraline
d. Fluoxetine
e. Paroxetine
f. Diazepam
g. Oxazepam
h. Other
i. I have not used medicines for depression and/or anxiety during this pregnancy

31. If you use medication for cardiovascular diseases, please list the medications you use.

a. Yes (Specify the medicine(s) you use for cardiovascular diseases during this pregnancy)
b. No

32. If you use medication for other disease(s), please list the medications you use

SafeStart Q2: Gestational weeks 13-26

INFORMATION ABOUT YOUR PREGNANCY

1. **What is your current gestational week?** (Specify)
2. **On average in a day, for how long do you feel nauseated or sick to your stomach?**
    - **a.** more than 6 hours
    - **b.** 4-6 hours
    - **c.** 2-3 hours
    - **d.** 1 hour or less
    - **e.** Not at all
3. **On average in a day, how many times do you vomit or throw up?**
    - a. More than 7 times
    - b. 5-6 times
    - c. 3-4 times
    - d. 1-2 times
    - e. Not at all
4. **On average in a day, how many times have you had retching or dry heaves without bringing anything up?**
    - a. More than 7 times
    - b. 5-6 times
    - c. 3-4 times
    - d. 1-2 times
    - e. Not at all
5. **Have you taken folic acid/folate during this pregnancy?**
    - a. Yes, before the pregnancy
    - b. Yes, before and during the pregnancy
    - c. Yes, during the pregnancy only
    - d. No
    - e. I don't recall

PREGNANCY-RELATED AILMENTS AND MEDICATION USE

6. **Have you experienced any pregnancy-related ailments during this pregnancy?**
    - a. Yes
    - b. No

Question 7 is only available if the woman ticks off any pregnancy-related ailments.

7. **Which of the ailments below have you experienced during this pregnancy?**
    - a. Nausea and vomiting
    - b. Heartburn
    - c. Constipation
    - d. Common cold and/or stuffy nose
    - e. Urinary tract infection
    - f. Other infections
    - g. Pain in the back, neck and/or pelvis
    - h. Headache
    - i. Sleep problems
    - j. Other (Specify)

Questions 8-15 are only available if the woman ticks off any of the related ailments above.

8. **Please select which medicine(s) you have used for nausea and/or vomiting during this pregnancy.**
    - a. Meclizine
    - b. Promethazine
    - c. Metoclopramide
    - d. Ondansetron

e. Prochlorperazine
f. Other
g. I have not used medicines for nausea and/or vomiting during this pregnancy

9. **Please select which medicine(s) you have used for heartburn during this pregnancy.**
   a. Alginic acid
   b. Magnesium hydroxide
   c. Ranitidine
   d. Omeprazole
   e. Other
   f. I have not used medicines for heartburn during this pregnancy
10. **Please select which medicine(s) you have used for constipation during this pregnancy.**
    a. Lactulose
    b. Ispaghula
    c. Bisacodyl
    d. Sennoside a+b
    e. Sodium picosulfate
    f. Klyx or Microlax
    g. Macrogol
    h. Other
    i. I have not used medicines for constipation during this pregnancy
11. **Please state which medicine(s) you have used for the common cold and/or stuffy nose during this pregnancy.**
    a. Xylometazoline
    b. Xylometazoline and ipratropium bromide
    c. Noscapine
    d. Ethylmorphine
    e. Codeine
    f. Bromhexine
    g. Guaifenesin
    h. Other
    i. I have not used medicines for the common cold and/or stuffy nose during this pregnancy
12. **Please select which medicine(s) you have used for urinary tract infections during this pregnancy.**
    a. Pivmecillinam
    b. Nitrofurantoin
    c. Trimethoprim (+Sulfamethoxazole)
    d. Amoxicillin
    e. Other
    f. I have not used medicines for urinary tract infections during this pregnancy
13. **Please select which medicine(s) you have used for pain in the back, neck and/or pelvis during this pregnancy.**
    a. Paracetamol
    b. Ibuprofen
    c. Naproxen
    d. Diclofenac
    e. Codeine and paracetamol
    f. Morphine
    g. Other
    h. I have not used medicines for pain in the back, neck and/or pelvis during this pregnancy
14. **Please select which medicine(s) you have used for headache during this pregnancy.**
    a. Paracetamol
    b. Ibuprofen
    c. Other

d. I have not used medicines for headache during this pregnancy

15. **Please select which medicine(s) you have used for sleep problems during this pregnancy.**
    a. Zopiclone
    b. Promethazine
    c. Alimemazine
    d. Zolpidem
    e. Other
    f. I have not used medicines for sleep problems during this pregnancy

CHRONIC DISEASES AND MEDICATION USE

16. **Do you have any chronic diseases?**
    a. Yes
    b. No

Question 17 is only available if the woman ticks off any chronic diseases.

17. **Please tick off whether you have any of the following chronic diseases listed below.**
    a. Asthma
    b. Allergies
    c. Hypothyroidism
    d. Rheumatic diseases (including rheumatoid arthritis and psoriatic arthritis)
    e. Type 1 or 2 diabetes
    f. Epilepsy
    g. Depression and/or anxiety
    h. Cardiovascular diseases
    i. Other (please specify which other disease)

Questions 18-26 are only available if the woman ticks off any of the related chronic diseases listed above.

18. **If you use medication for asthma, please select the medications you use.**
    a. Salbutamol
    b. Terbutaline
    c. Salmeterol
    d. Fluticasone
    e. Budesonide
    f. Ciclesonide
    g. Ipratropium bromide
    h. Other
    i. I have not used medicines for asthma during this pregnancy
19. **If you use medication for allergies, please select the medications you use.**
    a. Desloratadine
    b. Cetirizine
    c. Ebastine
    d. Loratadine
    e. Fexofenadine
    f. Nasal spray and/or eye drops only
    g. Other
    h. I have not used medicines for allergies during this pregnancy
20. **If you use medication for hypothyroidism, please select the medications you use.**
    a. Levothyroxine
    b. Liothyronine
    c. Combination of Levothyroxine and Liothyronine
    d. Other
    e. I have not used medicines for hypothyroidism during this pregnancy
21. **If you use medication for rheumatic diseases, please select the medications you use.**
    a. Ibuprofen

b. Paracetamol
c. Phenazone + codeine
d. Tramadol
e. Prednisolone
f. Methotrexate
g. Biological medications (given as injections)
h. Other
i. I have not used medicines for rheumatic diseases during this pregnancy

22. If you use medication for type 1 or 2 diabetes, please select the medications you use.

a. Insulin
b. Metformin
c. Glimepiride
d. Sitagliptin
e. Other
f. I have not used medicines for type 1 or 2 diabetes during this pregnancy

23. If you use medication for epilepsy, please select the medications you use.

a. Carbamazepine
b. Oxcarbazepine
c. Valproic acid
d. Lamotrigine
e. Topiramate
f. Levetiracetam
g. Other
h. I have not used medicines for epilepsy during this pregnancy

24. If you use medication for depression and/or anxiety, please select the medications you use.

a. Escitalopram
b. Citalopram
c. Sertraline
d. Fluoxetine
e. Paroxetine
f. Diazepam
g. Oxazepam
h. Other
i. I have not used medicines for depression and/or anxiety during this pregnancy

25. If you use medication for cardiovascular diseases, please list the medications you use.

a. Yes (Specify the medicine(s) you use for cardiovascular diseases during this pregnancy)
b. No

26. If you use medication for other disease(s), please specify the medications you use.
