# Supplementary material for: Impact of a primary care pharmacist consultations on pregnant women’s medication use: the SafeStart intervention study linked to a national prescription database
Source: Int J Clin Pharm. 2023 May 8;45(4):893–902. doi: 10.1007/s11096-023-01577-x (PMC10366231; doi:10.1007/s11096-023-01577-x)
Supplement: Supplementary file 2 — Supplementary file2 (PDF 186 KB) [file 11096_2023_1577_MOESM2_ESM.pdf]

Supplementary file 2: Overview of conducted pharmacist consultations, topics and pregnancy-related conditions addressed during the consultation.

	**Value**<br>Mean (range) or n	%
**Pregnancy week when receiving the pharmacist consultation**	9 (4-17)	
**Number of pharmacist consultations**		
At the pharmacies	37	*35.9*
On the phone	66	*64.1*
**Topics addressed during the consultation***		
General information about medications	32	*31.1*
Advice and treatment of pregnancy-related conditions	61	*59.2*
Need of medications	9	*8.7*
Negative attitudes and anxiousness about medication use	9	*8.7*
*Other topics related to medication use***	18	*14.5*
Need of referral to her GP	2	*1.9*
No topics addressed	12	*11.7*
**Pregnancy related conditions addressed during the consultation***		
Nausea and vomiting	49	*47.6*
Constipation	24	*23.3*
Heartburn	17	*16.5*
Cold/stuffy nose	21	*20.4*
Headache	14	*13.6*
Pain in general	11	*10.7*
*Other pregnancy related conditions****	10	*9.7*

n= Number of women

*One women can address serval topics and pregnancy related conditions

**Other topics related to medication use as anxious about the effect of the medication on the child and low adherence to regular medication

****Other pregnancy related conditions with below 10 cases includes sleeping problems, dizziness, and fatigue*
