# Supplementary material for: Impact of a primary care pharmacist consultations on pregnant women’s medication use: the SafeStart intervention study linked to a national prescription database
Source: Int J Clin Pharm. 2023 May 8;45(4):893–902. doi: 10.1007/s11096-023-01577-x (PMC10366231; doi:10.1007/s11096-023-01577-x)
Supplement: Supplementary file 3 — Supplementary file3 (PDF 195 KB) [file 11096_2023_1577_MOESM3_ESM.pdf]

Supplementary file 3: Overview of women with filled prescriptions as registered in the Norwegian Prescription Database, categorized after ATC-codes for, three months before pregnancy, 1st, 2nd, 3rd trimester, and three months post-partum.

ATC-code*	Three months before pregnancy n *(%)*	1st trimester n *(%)*	2nd trimester n *(%)*	3rd trimester n *(%)*	Three months post-partum n *(%)*
**A -** *Alimentary tract and metabolism*					
I	21 *(20.4)*	29 *(28.2)*	24 *(23.3)*	22 *(21.4)*	17 *(16.5)*
C	29 *(23.0)*	37 *(29.4)*	34 *(26.9)*	35 *(27.8)*	38 *(30.2)*
**B** - *Blood and blood forming organs*					
I	13 *(12.6)*	15 *(14.6)*	16 *(15.5)*	12 *(11.7)*	7 *(6.8)*
C	18 *(14.3)*	21 *(16.7)*	26 *(20.6)*	24 *(19.0)*	23 *(18.3)*
**G -** *Genito-urinary system and sex hormones*					
I	32 *(31.1)*	37 *(35.9)*	23 *(22.3)*	26 *(25.2)*	31 *(30.1)*
C	44 *(34.9)*	51 *(40.5)*	48 *(38.1)*	45 *(35.7)*	58 *(46.0)*
**H -** *Systemic hormonal preparations*					
I	15 *(14.6)*	16 *(15.5)*	13 *(12.6)*	10 *(9.7)*	15 *(14.6)*
C	24 *(19.0)*	28 *(22.2)*	26 *(20.6)*	26 *(20.6)*	27 *(21.4)*
**J** - *Antiinfectives for systemic use*					
I	36 *(34.9)*	41 *(39.8)*	36 *(34.9)*	29 *(28.2)*	35 *(33.9)*
C	36 *(28.6)*	41 *(32.5)*	47 *(37.3)*	39 *(30.9)*	48 *(38.1)*
**N -** *Nervous system*					
I	18 *(17.5)*	18 *(17.5)*	14 *(13.6)*	16 *(15.5)*	15 *(14.6)*
C	28 *(22.2)*	35 *(27.8)*	31 *(24.6)*	32 *(25.4)*	30 *(23.8)*
**R -** *Respiratory system*					
I	37 *(35.9)*	36 *(34.9)*	32 *(31.1)*	32 *(31.1)*	30 *(29.1)*
C	35 *(27.8)*	39 *(30.9)*	39 *(39.9)*	38 *(30.2)*	37 *(29.4)*
**Total**					
I	48 *(46.6)*	58 *(56.3)*	46 *(44.7)*	41 *(39.8)*	50 *(48.5)*
C	57 *(45.2)*	62 *(49.2)*	65 *(51.6)*	60 *(47.6)*	69 *(54.8)*

ATC-code= Anatomical Therapeutic Chemical Classification System, **n**= number of women, **I**= intervention group, **C**= control group

**ATC-code P (Antiparasitic products, insecticides and repellents), S (Sensory organs), and V (Various) is not included in this table as numbers of prescriptions in total were below 20 in the defined time period.*
