# Supplementary material for: Impact of a primary care pharmacist consultations on pregnant women’s medication use: the SafeStart intervention study linked to a national prescription database
Source: Int J Clin Pharm. 2023 May 8;45(4):893–902. doi: 10.1007/s11096-023-01577-x (PMC10366231; doi:10.1007/s11096-023-01577-x)
Supplement: Supplementary file 4 — Supplementary file4 (PDF 284 KB) [file 11096_2023_1577_MOESM4_ESM.pdf]

Supplementary file 1: Baseline characteristics of the study population compared to the general birthing population in Norway.

CHARACTERISTICS	n	Study population (n=229) Value (Median, range or %)	General birthing population in Norway Value (Median, range or %)
**Maternal age (years)**		31 (21-41)	31**
**Relationship status**			
Married/co-habitant	221	96.5	93.6**
**Higher education**			
Yes	194	84.7	51.5***
**Employment status**			
Employed	196	85.6	86.4****
**Primigravida**			
Yes	125	54.6	42.4**
**Folic acid supplement before/during pregnancy**			
Yes	226	98.7	33.8**

SD= standard deviation, **PUQE score**= Pregnancy Unique Quantification of Emesis score

***Other** chronic conditions includes ADHD, cardiovascular disease, Chronic fatigue syndrome, crohn`s disease, eczema, endometrioses, epilepsy, fibromyalgia, high cholesterol, hyperthyroidism, irritable bowel syndrome, mental disorders, migraine, multiple sclerosis, polycystic ovary syndrome, psoriasis, rheumatic diseases, sarcoidosis, and ulcerative colitis.

**Data from the Norwegian Medical Birth Registry for 2018

***Data from Statistics Norway, women aged 20–39 in 2018

****Data from Statistics Norway, women aged 25–39 in 2018
